# The Role of PPAR*γ* Receptors and Leukotriene B_4_ Receptors in Mediating the Effects of LY293111 in Pancreatic Cancer

**DOI:** 10.1155/2008/827096

**Published:** 2009-01-27

**Authors:** Thomas E. Adrian, Rene Hennig, Helmut Friess, Xianzhong Ding

**Affiliations:** ^1^Department of Physiology, Faculty of Medicine and Health Sciences, United Arab Emirates University, Al Ain, United Arab Emirates; ^2^Department of Surgery, Feinberg School of Medicine, Northwestern University, Chicago, IL 60611-3008, USA; ^3^Department of Surgery, Technische Universitaet Muenchen, 81675 Munich, Germany

## Abstract

Pancreatic cancer is a devastating disease in which current therapies are inadequate. Separate lines of research have identified the 5-lipoxygenase/leukotriene B_4_ receptor pathway and the PPAR*γ* pathway as potential targets for prevention or treatment of this disease. LY293111 was originally designed as a potent leukotriene B_4_ receptor antagonist for treatment of inflammatory conditions. LY293111 was also known to have inhibitory effects on 5-lipoxygenase, which is upstream of the production of leukotrienes. LY293111 was shown to have potent anticancer effects in pancreatic cancer and several other solid malignancies, where it caused cell cycle arrest and marked apoptosis. Subsequently, it came to light that LY293111 exhibited PPAR*γ* agonist activity in addition to its effects on the 5-lipoxygenase pathway. This raises the question of which of the two targets is of greatest importance with regard to the anticancer effects of this agent. The evidence to date is not conclusive, but suggests that the effects of LY293111 may be mediated by both LTB_4_ receptors and PPAR*γ*.

## 1. INTRODUCTION

Pancreatic Cancer is a devastating disease with more than 80% of all patients presenting with surgically inoperable tumors. It remains the fourth leading cause of cancer death in both men and women in the USA. The median survival is usually less than six months even with the addition of chemotherapy [1–3]. Surgical resection is the only effective treatment option, but there are few long-term survivors even after apparent curative resection [1–3]. Alternative effective treatment strategies are desperately needed for this disease.

### 1.1. Fatty acids and human cancer

Epidemiological and animal studies show that a high fat consumption is associated with a higher incidence and growth of tumors at several specific organ sites including breast, pancreas, and prostate [4–11]. Recent studies indicate that diets containing a high proportion of polyunsaturated omega-6 fatty acids (n−6 FA), such as linoleic acid (the precursor of arachidonic acid) are associated with a more advanced disease stage at the time of diagnosis of several kinds of cancer [4–6, 8, 10, 11]. In contrast, long-chain n−3 fatty acids, such as docosahexaenoic acid and eicosapentaenoic acid (EPA) inhibit the growth and metastasis of several cancers including pancreatic cancer [7, 9]. Omega 3 fatty acids inhibit tumor growth by a number of mechanisms including suppression of COX-2 expression and, for EPA at least, the alternative substrate produces different cyclooxygenase (PGE_3_) and lipoxygenase (LTB_5_) products that have anti-inflammatory and anticancer effects.

### 1.2. Eicosanoid pathways

Arachidonic acid is a substrate for three distinctively different enzymatic pathways. Among them, prostaglandin endoperoxide synthases (cyclooxygenases) catalyze the committed step that leads to prostaglandin biosynthesis [12–14]. The second pathway is the epoxygenase pathway that appears to have no role in cancer. The third pathway for metabolizing arachidonic acid, the lipoxygenase pathway catalyzes the incorporation of one oxygen molecule into polyunsaturated fatty acids to yield a 1-hydroperoxy-2, 4-trans, cis-pentadiene product [14–16]. Mammalian lipoxygenases possess regiospecificity during interaction with substrate, and on this basis have been designated as arachidonate 5-, 12-, and 15-lipoxygenase (5-LOX, 12-LOX, and 15-LOX) [14–16]. The three distinct enzymes insert oxygen at carbon 5, 12 or, 15 of arachidonic acid, and the primary product is 5S-, 12S-, or 15S-hydroperoxyeicosatetraenoic acid (5-, 12-, or 15-HPETE), which can be further reduced by glutathione peroxidase to hydroxy forms (5-, 12-, 15-HETE), respectively [14–16]. 5-LOX is noteworthy because it is the only pathway that can turn arachidonic acid into leukotrienes [15, 17]. The activity of 5-LOX is dependent upon a second factor termed 5-LOX-activating protein (FLAP) [15, 17]. Considerable effort has been expended by the pharmaceutical industry to produce inhibitors of FLAP, 5-LOX as well as leukotriene antagonists, because the 5-LOX products, leukotrienes (LTB_4_, LTC_4_, LTD_4_, and LTE_4_) have been implicated as mediators of inflammation and immediate hypersensitivity reactions, in particular, human bronchial asthma [18, 19].

### 1.3. Leukotriene receptor antagonists and the development of LY293111

The pharmaceutical industry has focused on several targets to suppress leukotriene activity in inflammatory conditions such as bronchial asthma [18, 19]. One approach is to directly inhibit 5-lipoxygenase activity, thereby blocking secretion of all leukotrienes. The most widely studied clinical inhibitor of 5-lipoxygenase is zileuton, which inhibits the active site of 5-lipoxygenase at concentrations that do not inhibit cyclooxygenase, 12-lipoxygenase, or 15-lipoxygenase [18–22]. Another avenue to inhibit leukotriene formation is via blocking FLAP activity, thus preventing cytoplasmic to membrane translocation and activation of 5-lipoxygenase [16–18]. MK-0591 is a widely used 5-lipoxygenase-activating protein inhibitor for biomedical research [16–18]. Even though it strongly inhibits 5-lipoxygenase activity and blocks leukotriene generation, its use in clinic is limited by marked side effects. The final pharmacological approach to block leukotriene activity is to selectively block the actions of LTB_4_ or the sulfidopeptide leukotrienes using specific receptor antagonists.

Several synthetic LTB_4_ receptor antagonists have been developed. Early compounds included SC-41930; ONO-4057, which was orally active; LY223982, a benzophenone dicarboxylic acid; and LY255283 a hydroxyacetophenone [23–27]. These latter two compounds from the Lilly Research Laboratories potently block LTB_4_ binding to its receptors within the nM range and inhibit the biological functions of LTB_4_ in vitro [28, 29]. Unfortunately, they showed poor oral bioavailability [28]. In 1995, investigators at the Lilly Research Laboratories reported a new LTB_4_ antagonist, LY293111. This compound is a novel derivative of LY255283, but is orally stable and more potent as an LTB_4_ receptor antagonist [28, 29]. Compared with other LTB_4_ receptor antagonists, LY293111 is superior at blocking the cellular functions induced by LTB_4_ [28, 30].

### 1.4. Inflammation, Cyclooxygenases, Lipoxygenases, and cancer

The epidemiological data show a clear and strong association between chronic inflammatory conditions and cancer development, even though the conditions causing inflammation may vary [31–36]. It can be due to chronic infection caused by a virus, bacteria, or parasite or it may be due to noninfective, physical, or chemical irritant [31–36]. For example, chronic infection with the bacterium *Helicobacter pylori* causes atrophic gastritis, which can lead to dysplasia and adenocarcinoma [37]. Hepatitis B and C viruses account for more than 80% of cases of hepatocellular carcinoma worldwide [34]. The inflammatory bowel diseases, ulcerative colitis and Crohn's disease, predispose to the development of cancers of the large bowel and/or terminal ileum, although a causative infectious agent has never been conclusively identified [38]. For noninfectious inflammation, chronic reflux of gastric acid and bile into the distal esophagus causes chemical injury and on the long-term can lead to Barrett's esophagus and eventually to esophageal adenocarcinoma [35]. Thus it is apparent that chronic inflammation is a common underlying theme in the development of many different malignancies.

Although the mechanisms for the association between inflammation and cancer are not fully understood, growth factors, cytokines, and chemokines released into inflammatory environment are associated with tumor development and progression [32, 36]. High concentrations of free radicals and nitric oxide can induce DNA damage and promote cancer development [32, 36]. Over the past decade, much attention has been paid on the role of cyclooxygenases in cancer development, specifically its inducible isoform, the cyclooxygenase 2 (COX-2) [39–41]. COX-2 is active within both inflamed and malignant tissues [37–39]. The expression of COX-2 and COX-2 metabolites increases during the multistage progression of tumors [39–41]. By metabolizing arachidonic acid to prostaglandins, COX-2 induces cellular resistance to apoptosis, modulation of cellular adhesion and motility, promotion of angiogenesis, and immunosuppression [42–47]. Epidemiological data has implicated COX-2 in the pathogenesis of a number of epithelial malignancies, especially colorectal cancer [48, 49]. Inhibition of the enzyme with COX inhibitors is associated with a dramatic reduction in the incidence, morbidity and mortality of colorectal cancer [48–51]. Recent attention has also been focused on the role of 5-LOX, 12-LOX, and 15-LOX in cancer [52–60]. In pancreatic cancer, activation of the 5-LOX and 12-LOX pathways enhances cancer cell proliferation, while the 15-LOX pathway is protective against cancer development [61–64].

### 1.5. The 5-lipoxygenase/leukotriene B_4_ pathway and cancer

Accumulating evidence suggests that the 5-LOX pathway has profound influence on the development and progression of human cancers [61–64]. 5-LOX is overexpressed in pancreatic cancer tissues but is not expressed in normal pancreatic ductal cells [65]. Furthermore, this pathway is already up-regulated in pancreatic intraepithelial neoplasias (PanINs), which are the precursor lesions of pancreatic adenocarcinoma [66]. Blockade of 5-LOX activity inhibits proliferation and induces apoptosis in pancreatic cancer cells both in vitro and in vivo [67–69]. Pancreatic cancer cells secrete LTB_4_ and LTB_4_ induces proliferation in these cells [62]. Two G-protein-coupled LTB_4_ receptors (BLT1 and BLT2) have been cloned and characterized. BLT1 and BLT2 are high- and low-affinity LTB_4_ receptors, respectively, and form a gene cluster in humans. Both BLT1 and BLT2 are up-regulated in pancreatic cancer tissues, and expression was seen in all of the tested pancreatic cancer cell lines [65, 70]. As with other proteins in the 5-LOX/LTB_4_ pathway, BLT1 and BLT2 are already up-regulated in pancreatic intraepithelial neoplasias (PanIN lesions) which are the precursors of pancreatic adenocarcinomas [70]. This suggests that they may be valuable targets for chemoprevention.

### 1.6. PPAR*γ* and pancreatic cancer

**Figure 1:**
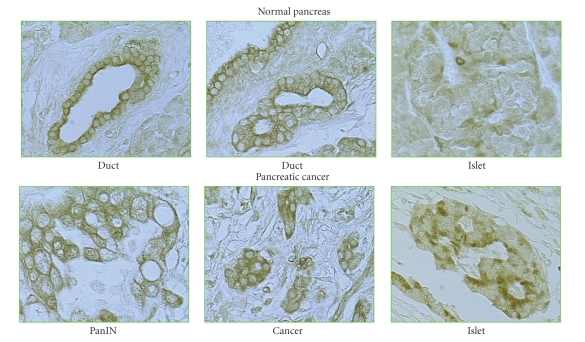
Immunocytochemistry of PPAR*γ* receptor in normal human pancreas and in a PanIN lesion and a pancreatic cancer. The PPAR*γ* receptor antibody shows a similar intensity of nuclear staining in normal ducts and in pancreatic cancer cells as well as cells in the PanIN lesion. In contrast, no staining is seen in normal islets, but nuclear staining is seen in islets from tissue adjacent to a cancer. These pictures are representative of eight samples of each tissue type. Staining was similar using antibodies from two different commercial sources.

Peroxisome proliferator activated receptor-*γ* (PPAR*γ*) is a member of the nuclear receptor superfamily of ligand-activated transcription factors. PPAR*γ* is expressed at high levels in adipose tissue and plays a central role in adipocyte differentiation and energy homeostasis. Recent studies have implicated PPAR*γ* in the pathogenesis of several human malignancies [71–74]. Previous studies have suggested that PPAR*γ* is up-regulated in pancreatic cancer [75]. Our own studies, employing two separate commercially available antibodies, show that PPAR*γ* is expressed in pancreatic cancer, but that expression in the cancer cells does not appear to be different from that in normal pancreatic ductal cells (Figure 1). In contrast, PPAR*γ* staining was seen in the islets surrounding cancers, but not in islet cells from normal pancreatic tissues obtained from multiorgan donors (Figure 1). In animal models, PPAR*γ* ligands have preventive effects against chemical carcinogenesis [76]. Several studies have shown that PPAR*γ* agonists, including the natural ligand 15-deoxy-Δ12,14-prostaglandin J2, and thiazolidinedione antidiabetic agents, such as citiglitizone and rosiglitizone, inhibit growth and induce apoptosis in pancreatic cancer [75, 77–81]. In contrast, one paper suggests induction of differentiation without apoptosis [82]. The apoptosis appears to be preceded by a morphological change to a more differentiated cell type which perhaps undergoes apoptosis when DNA repair turns out to be not possible [80]. In some studies, PPAR*γ* agonists also block invasion and angiogenesis [83, 84]. However, this is controversial since PPAR*γ* agonists induce secretion of vascular endothelial growth factor, which would have a promoting effect on metastatic tumor growth [85].

### 1.7. LY293111 and cancer

As might be expected from the growth-stimulatory effects of LTB_4_ in pancreatic cancer, the LTB_4_ receptor antagonist, LY293111 inhibits cancer growth and induces apoptosis both in vitro and in vivo [86–89]. LY293111 inhibits proliferation and induces apoptosis in a wide range of pancreatic cancer cell lines as well as cells of other tumor types, such as breast, prostate, and colon cancer cells [86–89]. These effects on growth and apoptosis are both time and concentration dependent, with effects seen at 100–500 nM in vitro [86, 87]. To confirm the involvementof LTB_4_ receptors in mediating the effect of LY293111 on human pancreatic cancer cell proliferation, another selective LTB_4_ receptor antagonist, U75302 was used in comparison with a selective LTD_4_ antagonist, LY171883 [86]. U75302 inhibits the proliferation of pancreatic cancer cells; but it is less potent than LY293111 as expected from the lower receptor affinity of this drug [28, 29, 90]. In contrast, the selective LTD_4_ antagonist, LY171883 had no significant effect on pancreatic cancer cell growth. LY293111 causes cell cycle arrest in the S phase of the cell cycle with suppression of expression of cyclin A, cyclin E, and cdk2. In parallel with growth inhibition, LY293111 induced apoptosis in all cancer cell lines tested [86, 87]. LY293111 induced dramatic morphological changes inhuman pancreatic cancer cells following a short period of treatment [86, 87]. The treated cells became rounded and exhibited membrane blebbing, chromatin condensation, and nuclear fragmentation, finally they were detached from the microplate. Induction of DNA fragmentation by LY293111 was confirmed by TUNEL assay (terminal deoxynucleotidyl transferase-mediated nick end labeling) and apoptosis was also established by annexin V binding [86, 87]. Apoptosis is triggered through the mitochondrial pathway, with a change in the ratio of proapoptotic proteins, such as Bax to antiapoptotic proteins, such as Bcl-2 and Mcl-1, release of cytochrome C, activation of caspase (but not caspase 8), and subsequent activation of the downstream caspase cascade with activation of caspase 3 and caspase 7 and cleavage of the caspase 3 substrate, poly ADP-ribose polymerase (PARP) [87].

LY293111 markedly slows down the growth of subcutaneous xenografts of human pancreatic cancer in athymic mice at a dose of 250 mg/kg/day [86]. To confirm the antipancreatic cancer effect of LY293111, pancreatic cancer cells with stable expression of enhanced green fluorescent protein (GFP) were orthotopically implanted into the duodenal lobe of the pancreas of athymic mice. Our data show that LY293111 significantly inhibits the growth of the orthotopically implanted pancreatic cancer cells in concert with blocking metastatic spread to the liver and other organs [91]. LY293111 also dramatically increased the number of TUNEL positive cells in pancreatic tumors harvested from the subcutaneous transplant experiments in athymic mice [86, 91].

### 1.8. LY293111 as a PPAR*γ* agonist

Following the disclosure of anticancer effects of LY293111, researchers at the Lilly Research Laboratories found that LY293111 is also a PPAR*γ* agonist [92, 93]. This finding was initially based on structural analysis and was supported by functional studies. The PPAR*γ* agonist activity of LY293111 is evidenced by its ability to induce adipogenic differentiation in vitro [92]. Normalization of circulating glucose levels by LY293111 in the ZDF rat diabetes model further suggests that LY293111 is an antidiabetic, PPAR*γ* agonist [92]. Further studies suggested that the anticancer effect of LY293111 might be mediated, at least in part, by PPAR*γ* [92, 93]. More extensive studies have subsequently shown that LY293111 is also an inhibitor of 5-lipoxygenase, although this effect is less potent than the LTB_4_ and PPAR*γ* targets.

### 1.9. Mechanisms by which LY293111 functions in cancer

**Figure 2:**
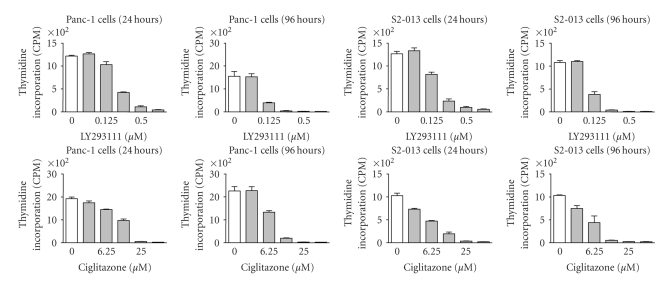
Comparison of the effects of different concentrations of LY293111 and ciglitazone on proliferation of two human pancreatic cancer cell lines (Panc-1 and S2-013) after 24 and 96 hours of treatment, measured by thymidine incorporation. LY293111 was approximately 50 times more potent than ciglitazone at inhibiting proliferation of both cell lines. Data shown is mean±SEM from four separate experiments.

**Figure 3:**
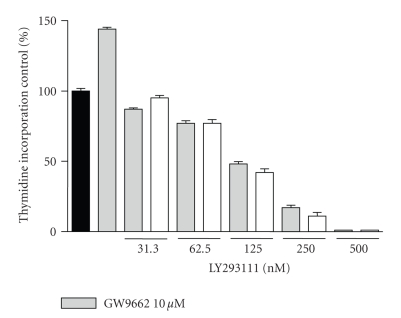
Effect of a PPAR*γ* receptor antagonist, GW9662 on the inhibition of proliferation induced by LY293111 in AsPC-1 human pancreatic cancer cells after 24 hours of treatment. These cells express both the PPAR*γ* receptor and LTB_4_ (BLT1 and BLT2) receptors. While GW9662 alone was able to significantly increase thymidine incorporation, it was not able to block the inhibitory effect of different concentrations of LY293111.

Since our findings suggest that all pancreatic cancer cells express both PPAR*γ* and BLT1, it is possible that the anticancer effects of LY293111 could be mediated by either receptor or both receptors. It has been reported that PPAR*γ* negative-expressing cancer cells are less responsive to LY293111-induced growth inhibition [92, 93]. However, there is also evidence in favor of BLT1 being the major target. Firstly, the effects of LY293111 on proliferation and apoptosis are extremely potent. A comparison between the effects of LY293111 and the PPAR*γ* agonist, ciglitazone is shown in Figure 2. As this figure shows, LY293111 is approximately 50 times more potent than ciglitazone in inhibiting the proliferation of Panc-1 and S2-013 human pancreatic cancer cells. LY293111 was also more potent than another PPAR*γ* agonist, rosiglitazone and the PPAR*α* agonist, WY-14643. The antiproliferative effects and induction of apoptosis are seen at 250 nM LY293111, which is much lower than the IC_50_ of the drug for PPAR*γ* receptors (~4 *μ*M) [92, 93]. Indeed, its effects on cancer cells are more potent than several PPAR*γ* agonists, including ciglitazone and rosiglitazone. Secondly, LY293111 is able to completely inhibit the effects of LTB_4_ on proliferation and MAP kinase activation in pancreatic cancer cells [62]. However, preliminary studies have shown that the antiproliferative effects of LY293111 in pancreatic cancer are not inhibited by the PPAR*γ* antagonist, GW9662 in vitro (Figure 3). Finally, data from our own studies and those of others show that PPAR*γ* agonists induce cell cycle arrest in the G0/G1 phase, whereas LY293111 induces S phase cell cycle arrest [82, 87]. It is even possible that anticancer effects of LY293111 might also be partially mediated by other unknown mechanisms. However, based on the current data, both the leukotriene B_4_ receptor and PPAR*γ* are likely to be involved in the antitumor activity of LY293111.

## 2. EFFECT OF LY293111 IN COMBINATION WITH OTHER AGENTS IN CANCER

Several studies have demonstrated that LY293111 enhances anticancer effects of gemcitabine, which is widely used as the standard therapy in pancreatic cancer patients in adjuvant and palliative treatment settings [89, 91, 94]. Gemcitabine only improves survival by a few weeks, but clinical data show improvement in the quality of life for pancreatic cancer patients. The effects of LY293111 in combination with gemcitabine were investigated in an orthotopic model of pancreatic cancer in athymic mice [91]. This model is superior to subcutaneous transplantation since it is less likely to modify the biological characteristic of pancreatic cancer cells, providing a favourable growth environment for them. It also allows easy monitoring of hepatic and lymph node metastasis with GFP stable expressing cells. In this model, animals without any treatment following implantation of GFP-expressing, S2-O13 pancreatic cancer cells developed end-stage disease with invasive cancer obstructing the duodenum and bile duct [91]. The animals develop liver, lung, and lymph node metastases and eventually peritoneal carcinomatosis with malignant ascites and cachexia [91]. Either gemcitabine or LY293111 alone significantly inhibited tumor growth and reduced the incidence of liver metastasis. However the combination of LY293111 and gemcitabine was significantly more effective than either treatment alone in blocking tumor growth [91]. Combined treatment also significantly relieved tumor-induced cachexia and maintained stable body weights compared with either drug alone, and also significantly decreased the incidence of biliary obstruction and metastasis [91]. These experimental results show that combined therapy of gemcitabine and LY293111 potently inhibits the growth and metastases of the very rapidly growing and aggressive pancreatic adenocarcinoma and suggest that it might be a valuable way for treatment of pancreatic cancer patients. LY293111 has also been shown to increase the effectiveness of gemcitabine in a colon cancer model [89]. The in vitro effects of LY293111 have been tested with other classical chemotherapeutic agents. The effects of the active metabolite of irinotecan, SN-38 or the active metabolite of capecitabine, 5′-DFUR were enhanced by LY293111 in multiple cell lines, including breast, bladder, and sarcoma cells [94].

## 3. CLINICAL TRIALS WITH LY293111

Three phase I clinical trials with LY293111 alone or in combination with gemcitabine or irinotecan have been reported. LY293111 was generally well tolerated [95–97]. The side effects were mild to moderate; the major ones gastrointestinal with diarrhea and pain. These initial phase I trials looked promising and LY293111 could be safely administered orally [95–97]. For example, in combination with gemcitabine, three patients had partial responses [96]. One had pancreatic cancer previously treated with gemcitabine, one with pancreatic cancer previously treated with 5-flurouracil and radiation, and one with non-small-cell lung cancer treated with one prior regimen. Two phase II trials have been completed and preliminary data reported in abstract form [98, 99]. One of these compared the combination of LY293111 with gemcitabine compared with gemcitabine with placebo in pancreatic cancer [98]. The second compared LY293111 with cisplatin and gemcitabine versus the placebo combined with the latter two drugs in patients with non-small-cell lung cancer [99]. Unfortunately, LY293111 did not improve progression-free survival in either of these two trials [98, 99].

## 4. CONCLUSIONS

LY293111 is an interesting compound that has biological effects on several different targets. It acts as an antagonist on LTB_4_ receptors, as a PPAR*γ* agonist and as a 5-lipoxygenase inhibitor. Indeed, some investigators have referred to LY293111 as a multiple eicosanoid pathway inhibitor [94, 99]. Since LY293111 has anticancer effects on multiple tumor types, the target involved in mediating the effects of the drug is clearly of interest. The evidence to date is not conclusive, but suggests that effects may be mediated by both LTB_4_ receptors and PPAR*γ*. Preliminary reports regarding the phase II clinical trials have unfortunately been disappointing. It remains unknown whether this compound will eventually find a use in the clinic for cancer therapy; however this recent clinical experience perhaps makes this unlikely now.
